# Bilateral improvement in age-related macular degeneration following unilateral Aflibercept injection

**DOI:** 10.1186/s12886-024-03795-x

**Published:** 2024-12-05

**Authors:** Muhammad Irfan Karamat, Hira Saleem, Minahil Khakwani, Abdullah Ahmed

**Affiliations:** 1 Department of Ophthalmology, Jinnah Hospital, Allama Shabbir Ahmad Usmani Road, Lahore, Punjab 54550 Pakistan; 2 Nishtar Medical University, Multan, Punjab 66000 Pakistan

**Keywords:** AMD, Aflibercept, Fellow Eye Effect, Anit-VEGF, Pakistan

## Abstract

**Background:**

The "fellow-eye effect" in anti-vascular endothelial growth factor (anti-VEGF) therapy is a rare phenomenon, particularly with aflibercept. This case report documents the first instance of this effect in Pakistan, highlighting its significance in a resource-limited setting where recent regulatory changes have restricted treatment options for age-related macular degeneration (AMD).

**Case presentation:**

A 62-year-old male presented with bilateral central vision loss due to neovascular AMD. Optical Coherence Tomography revealed serous subretinal fluid in both eyes. Due to financial constraints, only the right eye received a single 2 mg intravitreal aflibercept injection. Remarkably, at the four-week follow-up, both eyes showed significant improvement. Visual acuity improved from 6/12 to 6/9 in the treated right eye and from 6/15 to 6/12 in the untreated left eye. OCT scans demonstrated bilateral resorption of subretinal fluid. At three months, complete fluid resorption was observed in both eyes, with visual acuity improving to 6/6 bilaterally.

**Conclusions:**

This case underscores the potential of the "fellow-eye effect" in anti-VEGF therapy, particularly with aflibercept, in treating bilateral AMD. It highlights a possible strategy for optimizing treatment regimens and reducing costs in resource-limited settings. However, it also raises concerns about systemic absorption and potential risks. The findings emphasize the need for further research on the pharmacokinetics of anti-VEGF agents, personalized treatment plans, and alternative therapies. This case is particularly significant in the context of Pakistan's recent ban on bevacizumab, underscoring the urgent need for accessible and affordable AMD treatments in developing countries.

## Introduction

Anti-vascular endothelial growth factor (anti-VEGF) agents have revolutionized the treatment of various ophthalmological conditions, including age-related macular degeneration (AMD), diabetic macular edema, and retinal vessel occlusion [1]. These agents work by inhibiting VEGF, a key factor in pathological angiogenesis and vascular permeability, thereby significantly improving patient outcomes and preserving vision.

One intriguing phenomenon observed with the use of anti-VEGF agents is the 'fellow-eye effect', where an intravitreal injection in one eye appears to influence the untreated contralateral eye. Despite its clinical relevance, this effect is sparsely documented in the published literature. The fellow-eye effect has been primarily reported with bevacizumab [2, 3] and ranibizumab [1, 3, 4], with several studies and case reports highlighting these occurrences. However, instances involving aflibercept are particularly rare. Only a limited number of cases demonstrating the fellow-eye effect with aflibercept have been documented in the literature [1], underscoring the need for further investigation and documentation to better understand the mechanisms and implications of this phenomenon.

## Case presentation

A 62-year-old male presented to the ophthalmology department of Jinnah Hospital in Lahore, Pakistan, with a two-month history of bilateral central vision loss, more pronounced in the right eye. The patient had no history of hypertension, diabetes, or other significant medical conditions. Blood sugar levels were normal and family history was negative for inherited eye disorders. He reported no smoking or alcohol consumption.

**Fig. 1:**
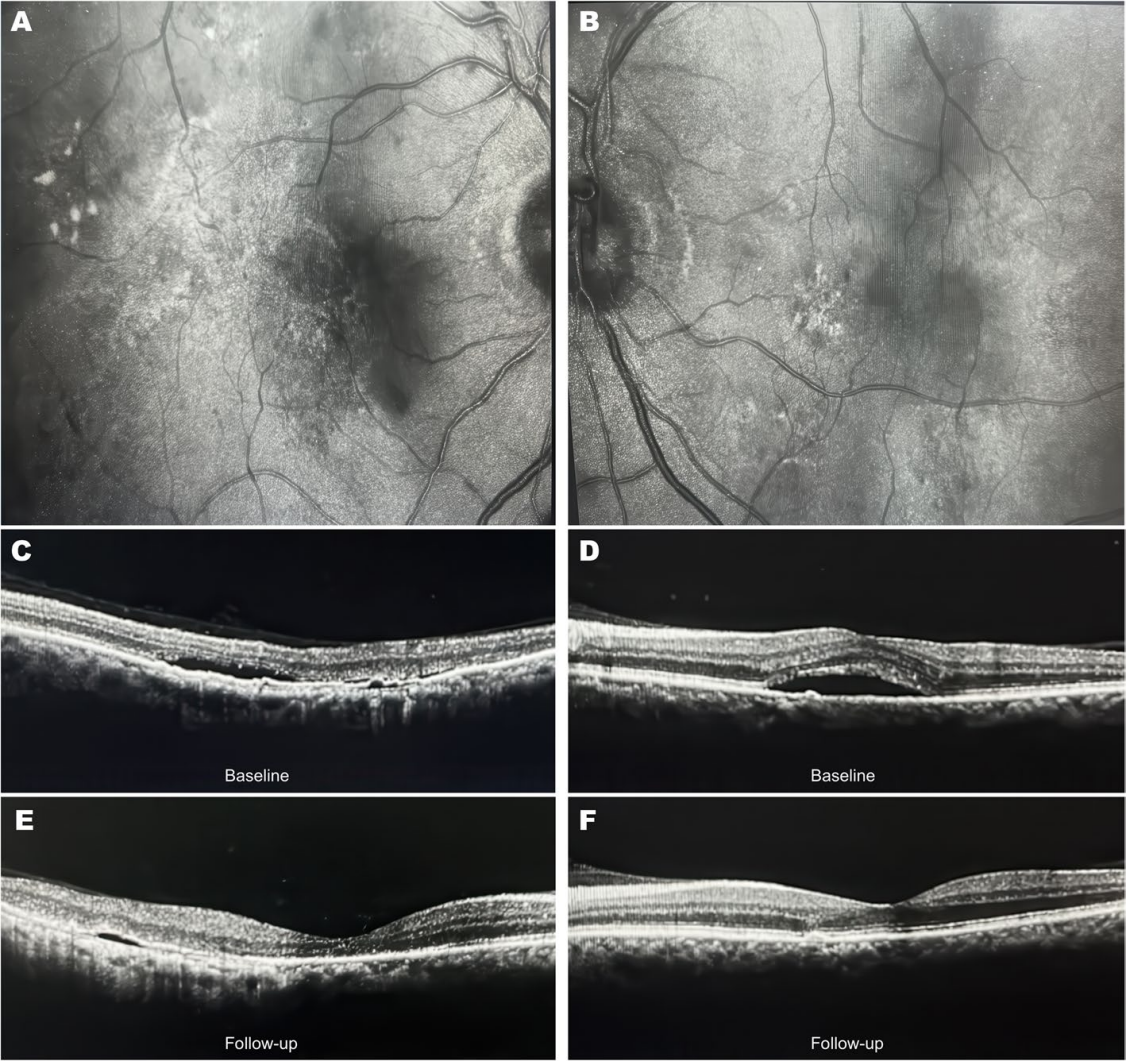
Fundus and OCT images of bilateral neovascular age-related macular degeneration. **A**, **B** Fundus images of right and left eyes showing hard exudates. **C**, **D** Baseline OCT images demonstrating subfoveal subretinal fluid in both eyes at baseline. **E**, **F** OCT images at 4-week follow-up. Treated right eye (**E**) and untreated left eye (**F**) show significant reduction of subretinal fluid and improvement in macular edema

On examination, visual acuity was 6/12 in the right eye and 6/15 in the left eye. Intraocular pressures were 12 mmHg and 14 mmHg in the right and left eyes, respectively. Pupillary light reflexes were intact bilaterally, and extraocular movements were full. Anterior segment examination was unremarkable. Slit-lamp examination revealed an altered macular reflex in the posterior segment in both eyes. Fundus imaging showed hard exudates in the outer plexiform layer and corrugation of retinal pigment epithelium, indicating choroidal neovascularization (Fig. 1A and B). Optical Coherence Tomography (OCT) performed using Nidek RS-3000 revealed serous subretinal fluid in the subfoveal region of both eyes (Fig. 1C and D). The right eye OCT also demonstrated minor pigment epithelial detachment. Based on these findings, the patient's advanced age, and absence of diabetic history, a diagnosis of neovascular age-related macular degeneration (AMD) was made.

The ophthalmologist recommended bilateral intravitreal aflibercept (EYLEA®, Bayer HealthCare, Berlin, Germany/Regeneron Pharmaceuticals Inc., Tarrytown, USA) injections. However, due to financial constraints, the patient opted for treatment only in the right eye.Following standard aseptic technique, 2 mg (0.05 mL) of aflibercept was injected into the vitreous cavity of the right eye. No immediate complications were observed, and the patient was scheduled for follow-up after four weeks.

**Fig. 2:**
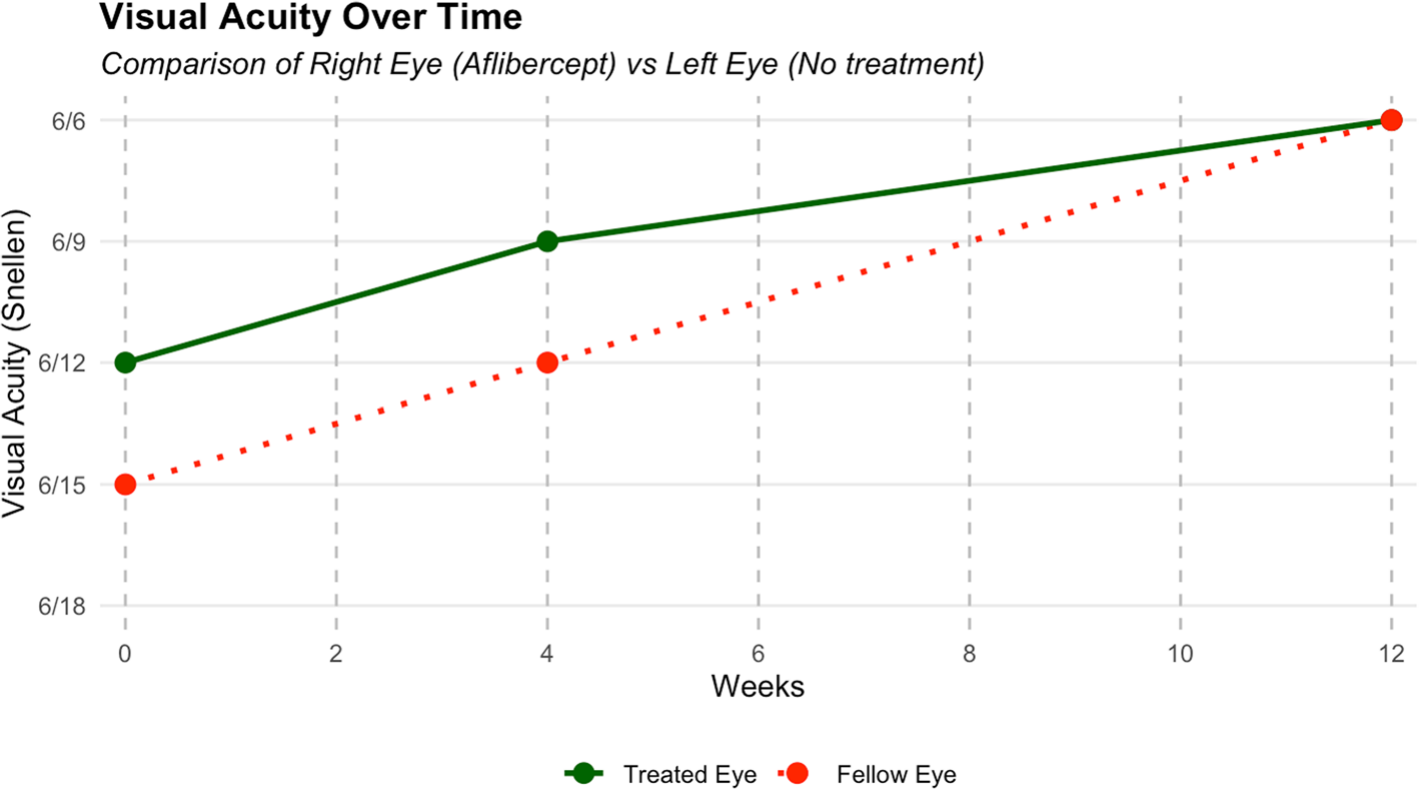
Visual acuity improvement over 12 weeks in treated (right eye, aflibercept) versus untreated (left eye)

At four weeks follow-up visit, the patient reported significant improvement in vision and reduction of central scotomas in both eyes. Repeat OCT scans showed significant resorption of subretinal fluid bilaterally (Fig. 1E and F). Visual acuity improved to 6/9 in the right eye and 6/12 in the left eye at four weeks follow up. At a subsequent three-month follow-up, complete resorption of subfoveal fluid was observed in both eyes. Visual acuity had further improved to 6/6 bilaterally and remained stable thereafter. Line graph demonstrating improvement in visual acuity is given in Fig. 2.

## Discussion

This case report presents a striking example of the "fellow-eye effect" observed with aflibercept in the treatment of neovascular age-related macular degeneration (AMD), with significant implications for clinical practice. Notably, this is the first documented case of this phenomenon in Pakistan, a country with limited healthcare resources, highlighting the need for further research in diverse populations.

Aflibercept functions by binding to VEGF-A, VEGF-B, and placental growth factor (PlGF), preventing their interaction with endothelial cell receptors. This blockade inhibits angiogenesis and reduces vascular permeability, ultimately improving retinal structure and function [5]. The drug's longer intravitreal half-life [6] compared to other anti-VEGF agents enables sustained therapeutic effects. Notably, bilateral improvement following unilateral injection occurs through several mechanisms. The high binding affinity to VEGF and prolonged half-life facilitate systemic absorption [7], while the neonatal Fc receptor (FcRn) aids in transporting these large IgG molecules across the retina-blood barrier. This systemic circulation allows aflibercept to reach and potentially benefit the untreated fellow eye through the blood-retinal barrier [8]. Additionally, anti-VEGF treatments may exhibit neuroprotective properties [5] beyond vascular effects, where reduced oxidative stress and inflammation in one eye could create a favorable environment for retinal cells in both eyes, particularly significant in AMD, which typically manifests as a bilateral disease.

The landscape of anti-VEGF therapy in ophthalmology is complex, particularly in resource-limited countries like Pakistan. The recent ban [9] on bevacizumab (Avastin) by the Drug Regulatory Authority of Pakistan (DRAP) in 2023 has further complicated the management of AMD, leading to devastating consequences for patients who suffered permanent vision loss due to lack of access to alternative treatments. Avastin, a low-cost drug, was a vital option for the average Pakistani citizen, particularly those from low- to middle-income backgrounds, who cannot afford the exorbitant prices of alternative treatments like aflibercept (Eylea) and ranibizumab (Lucentis). The "fellow-eye effect" offers a potential strategy for optimizing treatment regimens, reducing costs, and minimizing complications, particularly beneficial in resource-limited settings. This approach enables personalized treatment plans through tailored dosing regimens and potentially reduced injection frequencies, based on careful monitoring of fellow eye responses. Clinicians might initially opt for less expensive anti-VEGF agents when the fellow eye shows positive outcomes, before transitioning to costlier options if necessary [10]. Systematic assessment protocols, including cost-effective monitoring techniques and optimized frequency of high-cost procedures like fluorescein angiography [11], help identify early changes while reducing long-term costs. Resource optimization can be achieved through multi-eye treatment strategies and shared resources, including bulk purchasing between healthcare facilities. Additionally, patient education about the fellow eye effect may enhance treatment adherence, while healthcare provider training facilitates more efficient treatment strategies that capitalize on this phenomenon.

The "fellow-eye effect", although beneficial, also raises concerns about systemic absorption and potential risks [1], including undefined effects on VEGF's role in the body, stroke pathogenesis, gastrointestinal perforations, hypertension, and renal damage. Furthermore, the "fellow-eye effect" is not always beneficial, as reports of retinal pigment epithelium tears in the contralateral eye after administration of anti-VEGF agents exist in the literature [1].

This case report presents a valuable opportunity to explore and delve deeper into the “fellow eye effect” of aflibercept treatment in AMD. However, as our findings are based on a single patient, the generalizability of the results is inherently limited. Further studies with larger patient cohorts are needed to validate these observations and establish standardized protocols for monitoring and managing bilateral effects of unilateral aflibercept treatment.

## Conclusion

In conclusion, this case report highlights the significance of the "fellow-eye effect" in anti-VEGF therapy for AMD and underscores the need for further research to optimize treatment strategies. The ban on Avastin and the subsequent reliance on costly alternatives have severe implications for the Pakistani population, emphasizing the importance of accessible and affordable healthcare. We recommend that policymakers and healthcare providers work together to ensure the availability of low-cost treatment options, explore alternative therapies, and prioritize patient-centered care. Furthermore, continued research on the pharmacokinetics of anti-VEGF agents and the "fellow-eye effect" will be crucial in developing personalized treatment plans, reducing costs, and improving outcomes for patients with AMD. By addressing these challenges, we can work towards a more equitable and effective management of AMD in Pakistan and beyond.

## Data Availability

No datasets were generated or analysed during the current study.

## Abbreviations

AMD: Age-related macular degeneration
Anti-VEGF: Anti-vascular endothelial growth factor
OCT: Optical coherence tomography
IgG: Immunoglobulin G
FcRn: Neonatal Fc receptor
DRAP: Drug regulatory authority of Pakistan
